# Using Amino-Labeled Nucleotide Probes for Simultaneous Single Molecule RNA-DNA FISH

**DOI:** 10.1371/journal.pone.0107425

**Published:** 2014-09-16

**Authors:** Reelina Basu, Lan-Tian Lai, Zhenyu Meng, Jun Wu, Fangwei Shao, Li-Feng Zhang

**Affiliations:** 1 School of Biological Sciences, Nanyang Technological University, Singapore, Singapore; 2 Division of Chemistry and Biological Chemistry, School of Physical and Mathematical Sciences, Nanyang Technological University, Singapore, Singapore; NIH, United States of America

## Abstract

Using amino-labeled oligonucleotide probes, we established a simple, robust and low-noise method for simultaneous detection of RNA and DNA by fluorescence *in situ* hybridization, a highly useful tool to study the large pool of long non-coding RNAs being identified in the current research. With probes either chemically or biologically synthesized, we demonstrate that the method can be applied to study a wide range of RNA and DNA targets at the single-cell and single-molecule level in cellular contexts.

## Introduction

A growing list of long non-coding RNAs (lncRNAs) are being discovered in the current biological research [1]. Many of them play important roles in nuclear architecture and epigenetic regulation. Therefore, it is important to study how these RNA transcripts interact with their DNA targets in the nuclear context. Simultaneous RNA-DNA fluorescence *in situ* hybridization (RNA-DNA FISH) is a highly useful tool for this research topic. However, an RNA-DNA FISH experiment is often difficult because the fragile RNA FISH signals or the RNA targets cannot survive the harsh treatments (high temperature, low pH) used in DNA FISH to denature the target DNA [2], [3].

**Figure 1:**
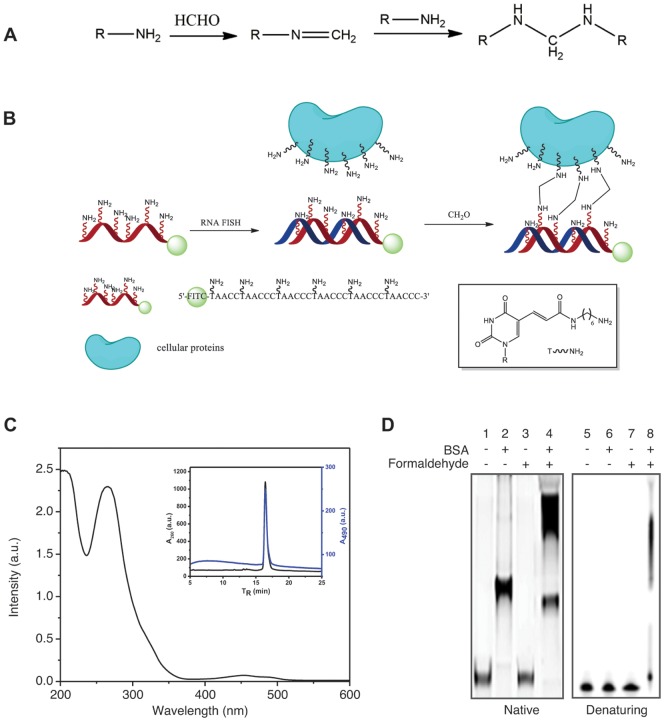
Scheme of amino-labeled DNA probes for formaldehyde fixation. (A) Chemical reactions of formaldehyde fixation. (B) Scheme of formaldehyde mediated crosslink between amino-labeled probe and cellular proteins in vicinity. (C) UV-vis absorption spectra of the oligonucleotide probe. *Insert*: HPLC purification trace of amino-labeled probes. (D) Electrophoretic mobility shift assay (EMSA) of formaldehyde mediated crosslink between the oligonucleotide probes and bovine serum albumin (BSA) on native (Lane 1 to 4) and denaturing PAGE (Lane 5 to 8).

In our previous study [2], we developed a method to overcome this difficulty by introducing immunostain into RNA FISH at signal detection steps. This, followed by a fixation step using formaldehyde (post-fixation), can efficiently protect the RNA signals from being damaged by the subsequent DNA FISH. The fixation step is used to crosslink the RNA signal with the cellular proteins in its close proximity, such that the RNA targets or the RNA FISH probes may still be damaged in the subsequent DNA FISH, but the RNA signals survive. For the fixation step to work, it is critical to include immunostain into RNA FISH, because formaldehyde-mediated crosslinking (Figure 1A) can only be efficiently applied to proteins, which contain lysine residues to offer the amino groups as crosslinking sites. Although this method provides robust protection for the RNA signals to survive the DNA FISH, the multiple steps of immunostain, which have to be included to preserve the RNA signals, make the method complicated, time-consuming and expensive. It also generates additional difficulties for the subsequent DNA FISH [2]. Furthermore, the immunostain unnecessarily amplifies the RNA signals and raises the background noise. To circumvent these disadvantages, in the current method, we introduced amino labels directly to the oligonucleotide probes for formaldehyde fixation between the probe and cellular proteins in RNA FISH (Figure 1B). Here, we show that amino-labeled nucleotide probes can be either synthesized chemically or enzymatically. RNA signals detected by amino-labeled nucleotide probes can be fixed by formaldehyde directly and survive the subsequent DNA FISH. The established method is simple and robust. We successfully applied the method to detect a variety of RNA targets including single RNA molecules.

## Materials and Methods

### Synthesis of amino-labeled oligonucleotide probes

Amino-labeled oligonucleotides were synthesized on an automated DNA synthesizer (Mermade 4, Bioautomation, USA) by using amino-dT phosphoramidite (Catalog #10-1039,Glen Research, USA). The standard coupling method was applied to incorporate amino-dT and all of the natural nucleosides. FITC labels were conjugated to DNA probes via solid phase phosphoramidite chemistry. FITC and amino dual labeled probes were deprotected by AMA treatment at room temperature for 2 hrs and were purified by reverse phase HPLC (Shimadzu, C18 column, 10 mm×250 mm, 0%–15% ACN over 50 mM triethylammonium acetate buffer, pH 7.0 over 30 minutes). To prepare Cy5-labeled oligonucleotide probes, DNA strands with or without amino-dT were first extended with a C6 alkyl amino linker at 5′-end on solid phase. Cy5 NHS ester (Catalog#23020, Lumiprobe, USA) was then coupled to amino terminal in the presence of 10 eq of DIPEA in anhydrous DMF for 2 hrs at room temperature. Cy5 labeled DNA strands were cleaved from resin and were deprotected in saturated ammonium hydroxide solution simultaneously for 12 hrs at 60°C. HPLC purification was then applied to the crudes. HPLC peaks with absorption of both DNA at 260 nm and Cy5 at 640 nm were collected. All of the oligonucleotide probes were confirmed by ESI-MS (Sangon, Shanghai, China).

### Electrophoretic mobility shift assay

20% native PAGE and 15% denature PAGE were applied to EMSA on duplex of probe and telomeric sequence (3 µL of 200 nM) in the presence or absence of Bovine serum albumin (Catalog #9048-46-8, Amersco, USA, 66.4 mg/mL) before and after formaldehyde fixation. Native and denature PAGE gels were run under 7 V for 7 hrs and 10 V for 4 hrs, respectively. All of the gels were then visualized by FITC fluorescence on a phosphorimager (Typhoon Trio, GE).

### Cell lines and culture

A transformed female mouse embryonic fibroblast (MEF) cell line [3], a human breast cancer cell line (HCC1937) (ATCC, Cat# CRL-2336) and a mouse embryonic stem cell line (J1) (ATCC, Cat# SCRC-1010) were cultured in DMEM medium with 10% FBS at 37°C in a 5% CO_2_ incubator.

### RNA and DNA FISH

Cells were either harvested by the cytospin method or directly cultured on cover slips. Cells were fixed and permeabilized before RNA or DNA FISH was carried out. The detailed experimental procedures have been described [2], [3]. In brief, for RNA FISH, slides were dehydrated, dried and hybridized with probes for 3 hours at 42°C in a dark and humid environment. For RNA-DNA FISH, after RNA FISH washing, slides were post-fixed prior to DNA FISH in 4% PFA for 15 min at room temperature and rinsed once with PBS. The slides were then denatured in 70% formamide, 2xSSC at 80°C for 10 min. Slides were dehydrated subsequently before denatured probes were applied for an overnight hybridization at 42°C in a dark humid environment.

For DNA FISH using the chromosome paint, two modifications were made to the denaturation conditions. The slides were treated with freshly made 0.1 M HCl 0.25% Tween for 1 min, washed with 2xSSC for 5 min before denaturing at 75°C for 10 min.

### Fluorescence *in situ* hybridization (FISH) probes

A FITC-labeled PNA probe (Panagene, Cat# F1009) was used in *Terra* RNA FISH. The amino-labeled oligonucleotide probe for detecting *Terra* was synthesized in-house. Nick translation labeled probes were prepared using nick translation kit (Roche, Cat# 10976776001). Nucleotide analogs used in probe labeling were Cy3-dUTP (Amersham, Cat# PA53022) and aminoallyl-dUTP (Jena Bioscience, Cat# NU-803S). Mouse *Xist* RNA was detected with Sx9 probe, a P1 DNA construct containing a 40 kb genomic fragment covering the mouse X inactivation centre (*Xic*) region. A ∼1 kb exon region of *HOTAIR* was PCR amplified from human genomic DNA using the primer pair: HOTAIR-F (5′-tgggagtgtgttttgttgga-3′) and HOTAIR-R (5′-gcacagaaaatgcatccaga-3′). A ∼1 kb exon region of *NEAT2* was PCR amplified from human genomic DNA using the primer pair: MALAT1-F (5′-cttcctgtggcaggagagac-3′) and MALAT1-R (5′-gcacctgcagagaaaaggag-3′). The PCR amplicons were cloned into plasmid vectors. Purified plasmid DNA was used as template DNA in nick translation to generate probes targeting the corresponding lncRNAs. Chromosome X paint was purchased from Cambio (Cat# 1189-XMF-02).

### Single molecule RNA FISH

For single molecule RNA FISH, an oligonucleotide probe set was designed by Stellaris Probe Designer (https://www.biosearchtech.com/) to target EGFP mRNA. The oligonucleotide probe set was labeled with one Cy5 dye at 5′ end. The corresponding amino-labeled probe set was synthesized with two or three amino labels in each oligonucleotide. The detailed experimental procedure of single molecule RNA FISH has been described [4].

### Lipofectamine transfection

Transformed MEF cells were transiently transfected with pEAK12GFP, an EGFP-expression plasmid, using Lipofectamine 2000 (Invitrogen Catalog #11668-019) according to the manufacturer's instructions.

### Microscopy and data analysis

Fluorescence images were collected on Eclipse Ti microscope (Nikon) using digital camera Clara Series model C01 (Andor) and deconvoluted using NIS Elements AR imaging software (Nikon).

To measure individual telomere DNA FISH signal intensity, images for multiple z-sections of a same slide were collected using the same exposure time. The images from the same z-section were merged and the sum fluorescent intensity of each telomere DNA FISH signal was measured using NIS Elements AR imaging software (Nikon).

To measure the surviving signal intensity of EGFP mRNA signals after DNA FISH, the total RNA signal intensity per cell was measured from the Cy5 channel. The EGFP protein fluorescent signal was used to mark the cell boundary. All fluorescent images from the two slides (the amino-labeled probe set and the regular probe set) were collected using the same exposure time for each fluorescent channel. The total EGFP protein florescent signal intensity per cell was also measured to normalize the experimental variation between the two slides and the EGFP expression level difference from cell to cell.

## Results

### Synthesis and *in vitro* characterization of amino-labeled oligonucleotide probe

We first synthesized an amino-labeled oligonucleotide probe to detect the telomeric repeat-containing RNA (*Terra*), a lncRNA transcribed from telomeric DNA regions [5]–[7]. The probe is a 36-nt DNA oligonucleotide with the sequence (TAACCC)_6_. An amino-modified thymine (amino-dT) was used to introduce an alkylamino group to the oligonucleotide probe with a density of at least one amino group every six nucleotides (Figure 1B). The oligonucleotide probes with dual labels of alkylamine and fluorophore were prepared in high yields after HPLC purification and exhibit UV-vis absorption features of both DNA and the fluorophore (Figure 1C). High volume preparation of the dual labeled probes can be readily achieved on high throughput DNA synthesis without major alterations in the protocols.

Formaldehyde cross-linking reactions of the amino-labeled oligonucleotide probes were first studied *in vitro*. By modifying on C5 of thymine, amino group with long alkyl chain can accommodate well in the major grooves of hybrid duplex DNA/RNA [8]. A 36 nt amino-labeled probe forms stable duplex with telomeric DNA, which shows one clear band on native PAGE (Figure 1D, lane 1). No intrastrand crosslinking is observed upon formaldehyde fixation, since no new bands and no alterations on the mobility of the original probe band have been observed in either native or denaturing PAGE (Figure 1D, lane 3 and 7). Unlike the crosslink agents such as iodo-uridine, with reaction site immediately next to nucleobases [9], the long alkyl linkage of amino-dT can send the crosslinking agent to a much wider reaction radius (>1 nm) around the probe and RNA targets. Crosslinking reactions between the amino-labeled probes and bovine serum albumin (BSA) are observed as smear bands with much slower mobility than the probes. More remarkably, the fact that formaldehyde fixation further retards the mobility of the protein bound probes (Figure 1D) indicates that crosslinking reaction may be extended to occur between the probes and non-bound protein which happens to be in the vicinal area. Similarly, during RNA FISH, the enlarged reaction radius by extending amino group with long linkage would increase the chance of the amino-labeled probes to be cross-linked with the surrounding proteins and hence to survive the subsequent DNA FISH.

### Simultaneous detection of *Terra* and telomere DNA

**Figure 2:**
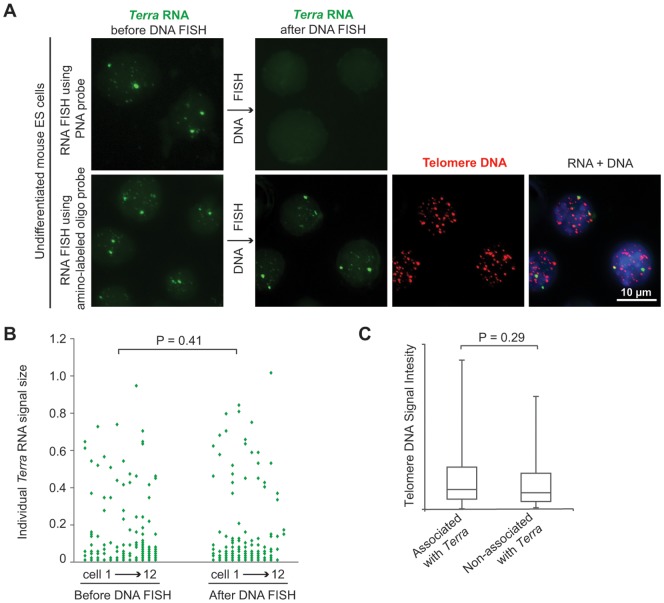
RNA-DNA FISH of *Terra* (RNA) and Telomere (DNA) using an amino-labeled oligonucleotide probe. (A) Either a FITC-labeled PNA probe (green) or an amino-labeled oligonucleotide probe (fluorescently labeled with FAM, green) was used to detect *Terra* in RNA FISH. Telomere DNA was detected by Cy3-labeled oligonucleotide probes (red) with the sequence of (GGGTTA)_6_. Nuclei were stained by DAPI (blue). (B) The *Terra* RNA signal number per nucleus and the signal sizes before and after DNA FISH were compared. A z-series of 3 µm thickness was collected. Images were deconvolved and merged. Individual *Terra* signals in each cell were computationally recognized by setting a fluorescence intensity threshold. The size of each *Terra* signal (µm^2^) was computed. *Terra* signals measured from one cell are plotted as discrete spots vertically at the x-axis. The P-value was calculated using Student t-test. (C) DNA FISH signal intensity of telomeres was measured to quantitate individual telomere length. The telomeres associated and non-associated with *Terra* were separated into two groups (n = 100 for each group). The data was presented in box-and-whisker plots. The P-value was calculated using Student t-test.

We then carried out *Terra* RNA FISH using the amino-labeled oligonucleotide probe and a peptide nucleic acid (PNA) probe. The PNA probe is chosen as the control, because the probe exhibits extraordinary thermal stability and is not susceptible to hydrolytic (enzymatic) cleavage [10]. In this case, the RNA signals of *Terra* were directly fixed by formaldehyde right after RNA FISH. All the immunostain steps were skipped. As shown in Figure 2A, the *Terra* signals detected by the PNA oligonucleotide probe were lost after DNA FISH despite the extraordinary thermal stability of the probe. In contrast, *Terra* signals detected by the amino-labeled probe survived the DNA FISH. The surviving *Terra* signals were bright and clear (Figure 2A). Several biological characters of *Terra* can be clearly recognized. Two major *Terra* RNA signals, which are significantly brighter and bigger than other *Terra* signals, can be detected in each nucleus in most of the mouse embryonic stem cells. In a given nucleus, not all the telomeres (DNA) are associated with *Terra* RNA. The *Terra* RNA transcripts locate in close proximity to telomeres, but some of the *Terra* signals do not overlap with telomere DNA. All of these characteristics of *Terra* are consistent with previous observations [2], [5]. It shows that the RNA-DNA FISH experiment worked well with the amino-labeled oligonucleotide probe. We also compared the RNA signal size and number per nucleus before and after DNA FISH (Figure 2B). The results show that the RNA signals were well protected from the damage of DNA FISH without significant loss in quantity and quality.

To date, the biological function of *Terra* remains largely unknown. Based on biochemistry evidence that *Terra* inhibits telomerase activity *in vitro*, it has been hypothesized that *Terra* associates with the long telomeres in a cell so that the telomerase activity is directed to concentrate on the short telomeres [6]. This hypothesis predicts that the telomeres associated with *Terra* are longer than those not associated with *Terra*. To confirm this prediction, we measured the DNA FISH signal intensity of individual telomeres to quantitate the telomere length. Our results show that the average length of telomeres associated with *Terra* is slightly longer than those non-associated (Figure 2C). However, the difference is not statistically significant. Our established RNA-DNA FISH method provided the first direct evidence of individual telomere length of telomeres associated and non-associated with *Terra*. We discuss this result in the discussion section.

### Detection of various RNA targets using amino-labeled probes generated by nick translation

**Figure 3:**
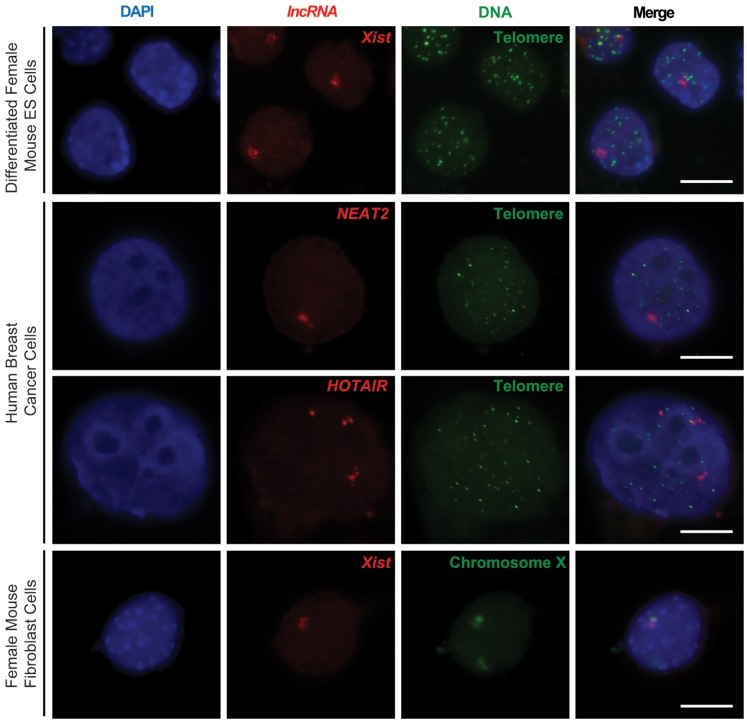
RNA-DNA FISH using amino-labeled probes generated by nick translation. Nuclei were stained by DAPI (blue). Three lncRNAs (*Xist*, *HOTAIR* and *NEAT2*) were detected using Cy3-labeled probes (red). Telomere DNA regions were detected using FITC-labeled oligonucleotide probes (green). Chromosome X territory was detected by FITC-labeled chromosome X paint (green) in DNA FISH. A scale bar of 10 µm is shown.

Synthetic oligonucleotide probe may not be an ideal choice in all RNA FISH experiments. It works well for *Terra*, because *Terra* carries a repetitive sequence, which provides multiple binding sites for the oligonucleotide probe within a single copy of RNA. For most RNA targets carrying non-repetitive sequences, the RNA signal detected by a single oligonucleotide probe would be too weak. In this case, a more suitable choice is to generate a random pool of fluorescently labeled short DNA fragments as a probe set by nick translation from a long DNA template [11]. In the nick translation reaction, alkylamino labels can be incorporated into the DNA fragments via an amino-labeled triphosphate nucleotide. To test this, we generated amino-labeled fluorescent probes using a commercially available amino-dUTP by nick translation (for details, see the ONLINE METHODS section) to target three well-known lncRNAs [12], [13]: the X-inactive specific transcript (*Xist*), HOX antisense intergenic RNA (*HOTAIR*) and noncoding nuclear-enriched abundant transcript 2 (*NEAT2*). We successfully detected all three lncRNAs in RNA-DNA FISH (Figure 3). The *Xist* RNA coats one X chromosome territory in each female somatic cell of mammals to transcriptionally inactivate the chromosome [13]. Coating of the *Xist* RNA can be visualized as a unique cloud signal in RNA FISH (the *Xist* cloud). In Figure 3, the *Xist* cloud signals were clearly detected simultaneously with telomere DNA or the chromosome X territory in RNA-DNA FISH. *HOTAIR* and *NEAT2* are over-expressed in cancer cells [14]. However, their associated DNA regions in the cancer nucleus are yet to be elucidated. In our experiments, we detected *HOTAIR* and *NEAT2* RNAs simultaneously with the telomeric DNA regions in human breast cancer cells (Figure 3). These results show that an amino-labeled nucleotide probe set can be generated by nick translation to detect various non-repetitive RNA targets in RNA-DNA FISH.

### Simultaneous detection of single mRNA molecule with DNA

**Figure 4:**
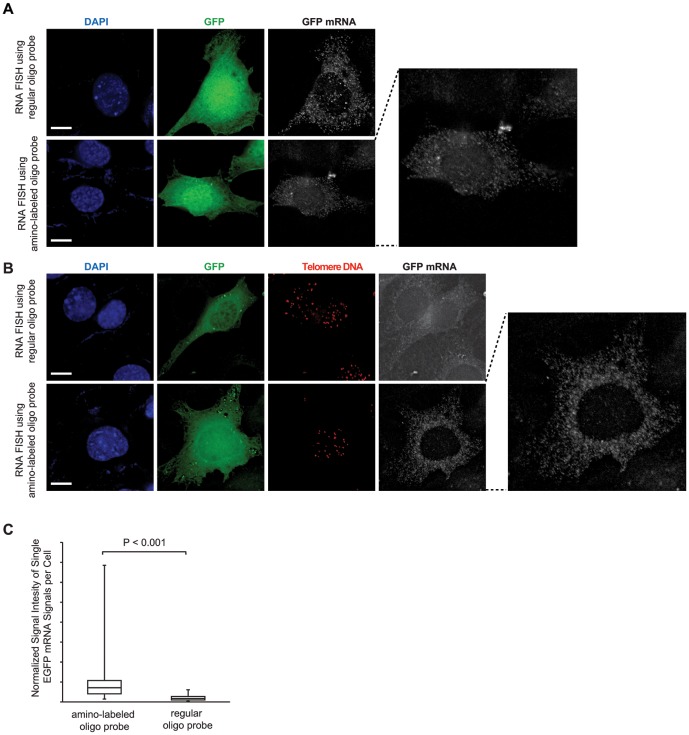
RNA-DNA FISH of the single mRNA molecules of EGFP and the telomeres (DNA). Telomere DNA was detected by Cy3-labeled oligonucleotide probes (shown in red). Nuclei were stained by DAPI (blue). The cells used were mouse fibroblast cells transiently transfected by an EGFP-expressing plasmid. The single mRNA molecules of EGFP were detected by a set of 20 oligonucleotides labeled by Cy5 (shown in grey). Two enlarged images of the single molecule RNA signals are shown on the right side. The images of the single molecule RNA signals are deconvolved single-layer z-sections. A scale bar of 10 µm is shown in each Dapi image. (A) RNA FISH only. (B) RNA-DNA FISH. (C) The total fluorescent signal intensity of the EGFP single mRNA molecule signals per cell (n = 50) measured from the Cy5 channel was normalized by the total EGFP fluorescent intensity per cell measured from the “green” channel. The data was presented in box-and-whisker plots. The P-value was calculated using Student t-test.

Using amino-labeled probe, the multiple immunostain steps involved in the previous method [2] are skipped, which not only simplifies the protocol, but more importantly, reduces the background noise. This enables us to apply the amino-labeled oligonucleotide probes to detect single RNA molecules. In single molecule RNA FISH [15], a set of synthetic oligonucleotide probes are designed to detect one target RNA. Previous studies have demonstrated that each mRNA molecule can be detected as a discrete pinpoint signal in the cytoplasm [16] and the image of single molecule RNA signals can be further processed by Laplacian of Gaussian (LoG) filter to obtain a sharper view [17]. As a proof of concept, we designed a set of 20 oligonucleotides to target the mRNA of the enhanced green fluorescent protein (EGFP) (Table 1) and successfully detected the single molecules of EGFP mRNA in the cytoplasm of mouse fibroblast cells transiently transfected by an EGFP-expressing plasmid (Figure 4A). We then prepared the amino-labeled probe set (Table 1) and showed that the similar pinpoint signals were obtained by the amino-labeled probe set in single molecule RNA FISH (Figure 4A). Finally, we carried out RNA-DNA FISH to simultaneously detect single molecule EGFP mRNA and telomere DNA. As shown in Figure 4B, the FISH signals of single EGFP mRNA molecule detected by the regular probe set suffered severe damage after DNA FISH, while the signals detected by the amino-labeled probe set survived DNA FISH. The surviving RNA signals remained as discrete pinpoint signals and only appeared in the cytoplasm of the EGFP-expressing cells. To quantitatively measure the surviving RNA signal quality, the total fluorescent signal intensity of the single EGFP mRNA signals per cell was measured. The EGFP protein fluorescent signal was used to mark the cell boundary. The total RNA signal intensity was divided by the total EGFP protein signal intensity to normalize the experimental variation from slide to slide and the EGFP expression level variation from cell to cell. Our results show that the surviving RNA signals detected by the amino-labeled oligo probes are significantly brighter than the surviving RNA signals detected by the regular oligo probes (Figure 4C).

**Table 1:** Sequences of amino-labeled probe set for single molecule RNA FISH.

Probe #	Sequence*
1	GtCCAGCtCGACCAGGAtGG
2	CtGAACTtGTGGCCGtTTAC
3	AGCtTGCCGtAGGTGGCAtC
4	GTGGtGCAGAtGAACTtCAG
5	CACtGCACGCCGtAGGtCAG
6	GACTtGAAGAAGtCGTGCtG
7	TGGACGtAGCCTtCGGGCAt
8	CTtGAAGAAGATGGtGCGCt
9	CGGGTCtTGTAGtTGCCGtC
10	GTtCACCAGGGTGtCGCCCt
11	CGAtGCCCtTCAGCtCGATG
12	ATGTtGCCGtCCTCCtTGAA
13	GTAGtTGTACtCCAGCtTGT
14	CAtGATAtAGACGtTGTGGC
15	AtGCCGtTCTTCtGCTTGtC
16	GCGGATCtTGAAGtTCACCt
17	CGCtGCCGtCCTCGAtGTTG
18	TGTGAtCGCGCtTCTCGtTG
19	GtCACGAACtCCAGCAGGAC
20	GtCCAtGCCGAGAGtGAtCC

*Cy5 fluorophore is attached to 5′-end of each probe. Thymines shown in lowercase letters are amino-labeled thymines.

## Discussion

Besides the post-fixation approach (using formaldehyde fixation to protect RNA FISH signals from the damage of the DNA FISH), an alternative approach for RNA-DNA FISH is to carry out the hybridization steps of the RNA FISH and the DNA FISH simultaneously. The presumption of this approach is that the RNA target survives the denaturation conditions used for DNA FISH. Two disadvantages should be considered for this approach. Firstly, it requires the FISH probes to be able to recognize the DNA and the RNA targets specifically, which is difficult to achieve in some cases, for example bi-directionally transcribed RNA transcripts coating the DNA region in *cis*. Secondly, a reliable denaturation condition, which is strong enough to denature the DNA target and mild enough to be harmless to the RNA target, may be difficult to find. These difficulties can be circumvented by our established method.

Using the established method, we show that the average telomere length of telomeres associated with *Terra* is slightly longer than those non-associated, but the difference is not statistically significant. This observation does not lend strong support to the hypothesis that *Terra* is associated with long telomeres to inhibit the telomerase activity and to concentrate the telomerase activity onto short telomeres [6]. Other than a negative regulator of the telomerase, *Terra* may play other functional roles. It has been noted that *Terra* expression sensitively responds to cellular stresses [5], [7]. Furthermore, *Terra* is not always associated with the telomere DNA [2]. Therefore, although *Terra* is transcribed from the telomere DNA, it may not always associate with telomere DNA in *cis* to function as a telomerase inhibitor. Future studies need to provide strong *in vivo* evidence to fully elucidate the functional roles of *Terra*. Nonetheless, our established method provided the first direct observation on the telomere length of individual telomeres associated and non-associated with *Terra*.

The name list of nuclear lncRNAs is growing rapidly in the current research. Many of these lncRNAs are involved in epigenetic regulations. The established method can be used to study the long list of regulatory lncRNAs and their DNA targets in the nucleus. In addition, the method may also be applied to clinical investigations. For example, the increased gene copy number of FGFR1 is used as a biomarker to predict the response of squamous-cell lung cancer patients to FGFR tyrosine kinase inhibitor in clinical trials [18]. However, high level of FGFR1 gene expression is not always directly related to the increased gene copy number [18]. Our RNA-DNA FISH method can provide direct observation at the single cell level for this topic.

In summary, we established a simple and robust method for simultaneous RNA-DNA FISH, which can be used to image RNA and DNA targets at the single-cell and single-molecule level. By conjugating alkylamino functionality to nucleotide probes via either oligonucleotide synthesis or nick translation, the current protocol can be applied to detect any potential RNA target and provide highly useful tools to study the large pool of nuclear IncRNAs.
